# Progress in Medicine

**Published:** 1897-06-26

**Authors:** 


### PROGRESS IN MEDICINE.

### DISEASES OF THE LUNGS.—TUBERCULOSIS.

(*Continued from page* 198.)

**Antiphthisin** was introduced about three years ago by Dr. E. Klebs. We are informed that it is made from a sterilised culture of tubercle bacilli after the entire removal of the latter. It is to be injected daily, subcutaneously, or intralaryngeally, or per rectum, or it may be applied externally if desired. The following conditions should be observed in using this remedy: (1) The patient must not be subjected to the first course of treatment for too short a space of time; it must last at least for four to eight weeks. (2) The treatment must be frequently repeated, say two or three times during the first year, and once or twice during the second year; and this carefully and conscientiously, even in cases which are apparently cured. It is advisable that several series of injections should be given, the dose being gradually increased; when the largest dose has been reached, it should be continued for some time, and subsequently the patient should proceed to the next series of injections. Each series of injections should last for ten to fourteen days.

The first series should be commenced by a daily dose of 0·1 to 0 5 c. cm., which should be increased daily by 0·1 c. cm., if there is no rise of temperature. Should it go up, the same dose should be continued until the temperature is stationary. The second series should be begun by a daily dose of 0·5 to 1·0 c. cm., increasing daily as in the first series. The third series should be begun with a dose of 1·0 to 2·0 c. cm, with a daily rise of 0·2 to 0·25 c. cm., until at length 2·0 to 4·0 c. cm. are injected. Larger quantities have been used without any injurious effect, but they are seldom necessary.

Rectal injections are as efficacious as those given subcutaneously. They are to be preferred in the case of sensitive patients and children, and also for a long course of treatment. Care must be taken that the very small doses are suitably diluted with a 0·2 per cent. solution of cresol in water, or with a 0·3 per cent. solution of carbolic acid. While many individual observers have reported results obtained with this remedy, we would especially draw attention to the report of the Parish Medical Society of New Orleans, a commission having been appointed to carry out the investigation; they arrived at the following conclusions, which they published early in 1896:—

(*a*) In Surgical Cases: A consideration of the three cases of improvement would certainly lead us to believe that antiphthisin has decided value, and we should commend its careful tentative employment in such cases, in conjunction with general measures and the usual appropriate operative treatment. The glandular case we consider especially encouraging. This case would seem to have required a most serious operation for the removal of the gland, with great uncertainty of ultimate benefit. The improvement under antiphthisin treatment would alone justify us in stating that we have in this remedy a most valuable aid in the management of such cases.

(*b*) In Medical Cases: In nearly every case the area of lung involved decreased, if it did not clear up entirely. Auscultation bore out the results of percussion, vesicular respiration replacing morbid breath-sounds in a greater or lesser degree. In cases which were classed as cured, the departure from health is only such as is due to the results of every continued pneumonic process. Secretion was diminished even in the cases marked only improved, and entirely absent in others. Bacteriological reports in most of the cases bore out the results obtained in physical and other examinations. The general condition of the patients improved in the large majority of cases, even in those whose physical examination did not show any great improvement. The use of the remedy was not attended with any danger to the patient. Finally, antiphthisin does seem to have curative, and not simply palliative, qualities.

**Paquin's Serum.**—An important communication from Dr. Paquin[13] himself deserves careful consideration. Regarding the globulicidal property of simple serum injections, he relates the results of his own experiments, which he describes, and which showed that in rabbits large injections into a vein resulted in rapid and pronounced oligocythæmia, with marked leucocytosis, and the same occurred in a human male. But this globulicidal property does not appear to be a constant factor of serum, for other similar experiments often failed to diminish the number of red cells in the same ratio, and often failed altogether. Now these experiments, he continues, argue not against serum therapy, but speak eloquently in favour of a better knowledge of this mode of treatment before its use. It does not show that it is a fatal remedy, but one to be used judiciously, with a proper understanding of all the points involved.

As to the explanation of the beneficial results of the serum, Paquin believes that these depend, in a great measure at least, on increased phagocytosis. Paquin records summaries of several cases treated by him, instances of both acute and chronic tuberculous disease being included. For these reports the reader is referred to his original article, but we may here include the summaries of one or two examples to illustrate the way in which the treatment is carried out in practice.

Acute tuberculosis. R. C. G., St. Louis, aged 60 years; Dr. Lloyd Simpson, St. Louis, was physician in charge; had three consultants, chief among them being Dr. J. R. Lemen, of St. Louis. Diagnosis of all was acute tuberculosis or tuberculosis pneumonia. Consolidation of the left lung almost complete, and extensive infiltration of the right. Prognosis, fatal termination. The patient at the beginning of treatment weighed between one hundred and fifty and one hundred and sixty pounds. Serum was continued about three months, more or less regularly every day, at doses ranging between 10 and 40 minims, after which it was administered irregularly for about two months. The patient suffered before the beginning of treatment with imperfect kidney secretion. The microscopical examination of the sputum was made first, Paquin states, by Dr. Ravold, bacteriologist of the city board of health and of the St. Louis Medical College, and later by Dr, George W. Cale, of the Woman's Medical College, his brother, a chemist, and himself, before, at the beginning, and during the course of the treatment, and in every instance, until after some weeks of treatment, the bacilli of tuberculosis were found in quantities. At the end of three months no bacilli were found in the sputum, and repeated analyses made since by some of the same analysts have failed to reveal them. The patient, who at the beginning of the treatment was in bed, picked up in flesh rapidly, and now weighs over two hundred and twenty pounds. He reports splendid health. Recent physical examinations confirm his opinion. He has recovered.

Miss G. A., St. Louis, aged 19 years; occupation, music and vocal student. Had had influenza in Memphis six years before; dry cough for a year; weighed one hundred and twenty-three pounds; hæmorrhages four years previous to examination (September 26th, 1895); larynx infiltrated; temperature, from 99·5 deg. to 101 deg. F.; coughing considerable, and expectoration of a yellowish matter, chiefly in the morning. Bacilli of tuberculosis quite numerous. Heart disease evidenced by regurgitation. Treatment began the last day of September, 1895. Injected very small doses on account of her heart condition—that is, 10 to 25 minims daily. At this time Miss A. weighs one hundred and thirty-five pounds. The bacilli of tuberculosis are now absent, and for two months there were but one or two in the field of bi-monthly examinations. Cough has almost entirely disappeared. Strength has been regained, and appetite is splendid. The patient is considered as having almost, if not quite, recovered, as the physical signs of infiltration and the first signs of breaking down which existed at the beginning of the treatment are absent. There still exists a slight expectoration, with micrococci.

[13] N.Y. Med. Journ., June 6, 1896.

